# Subclinical left ventricular myocardial dysfunction in patients with obstructive sleep apnea syndrome: insights from noninvasive left ventricular myocardial work analysis

**DOI:** 10.1186/s12872-022-03006-9

**Published:** 2022-12-19

**Authors:** Shan Jin, Xueyan Ding, Dichen Guo, Yunyun Qin, Weiwei Zhu, Zhiling Zhao, Xiheng Guo, Yidan Li, Xiuzhang Lu, Qizhe Cai

**Affiliations:** 1 Department of Echocardiography, Heart Center, Beijing Chao-Yang Hospital, Capital Medical University, 8 Gongren Tiyuchang Nanlu, Chaoyang District, Beijing, 100020 China; 2 Department of Respiratory and Critical Care Medicine, Beijing Chao-Yang Hospital, Capital Medical University, Beijing, 100020 China

**Keywords:** Myocardial work, Pressure–strain loops, Obstructive sleep apnea syndrome, Speckle tracking echocardiography

## Abstract

**Background:**

Obstructive sleep apnea syndrome (OSAS) is associated with various cardiovascular diseases and has aroused public concern. Early detection for declining myocardial function is of great significance. This study was aimed at noninvasively evaluating the subclinical left ventricular (LV) myocardial dysfunction with LV pressure–strain loop (PSL) in patients with OSAS having normal LV ejection fraction.

**Methods:**

We enrolled 200 patients with OSAS who visited the Beijing Chaoyang Hospital between February 2021 and December 2021. According to the apnea–hypopnea index (AHI), patients were divided into mild, moderate, and severe groups. The global longitudinal strain (GLS) of the left ventricle was analyzed by two-dimensional speckle tracking echocardiography. The LV PSL was used to assess global work index (GWI), global constructive work (GCW), global waste work (GWW), and global work efficiency (GWE), and comparisons were made among groups.

**Results:**

GLS was significantly lower in the severe group than in mild and moderate group. GWI, GCW, and GWE were lower in the severe group than in mild and moderate groups. GWW was significantly higher in the severe group than in the mild group. GLS, GWI, and GWE were moderately correlated with AHI (Spearman’s ρ = −0.468, −0.321, and −0.319, respectively; *P* < 0.001), whereas GCW and GWW showed a weak correlation with AHI (Spearman’s ρ = −0.226 and 0.255 respectively; *P* < 0.001). Multiple regression analyses revealed AHI was independently associated with GWI after adjusting for SBP, GLS, e’, etc. AHI was independently associated with GCW after adjusting for SBP, GLS, etc.

**Conclusions:**

The LV PSL is a new technique to noninvasively detect myocardial function deterioration in patients with OSAS and preserved LV ejection fraction. Increased severity of OSAS was independent associated with both decreased GWI and GCW.

**Supplementary Information:**

The online version contains supplementary material available at 10.1186/s12872-022-03006-9.

## Background

Obstructive sleep apnea syndrome (OSAS), which is characterized by recurrent reduction or cessation of breathing during sleep, has aroused extensive attention due to its high prevalence [1] and proven association with many cardiovascular diseases, such as atrial fibrillation, heart failure, hypertension, pulmonary hypertension, and stroke [2]. Intermittent hypoxia is the main mechanism behind cardiovascular complications. Early detection for declining myocardial function is of great significance.

Presently, left ventricular (LV) ejection fraction (LVEF) and two-dimensional speckle tracking echocardiography (2D-STE) are widely used to assess cardiac systolic function; however, they both have limitations in evaluating patients with OSAS. It is difficult to detect subtle changes in myocardial function at the early stage using LVEF [3]. Conversely, although 2D-STE—as a technique reflecting the length change of the ventricular myocardium—can detect a decline in subclinical myocardial function in patients with OSAS with preserved LVEF [4], this technique is load stress-dependent, and increased afterloads can lead to underestimation of systolic function [5].

LV pressure–strain loop (PSL) is a very promising new technique derived from 2D-STE to detect the slight subclinical changes. It takes both myocardial deformation and afterload into consideration and has been used in many diseases because of its accuracy and reproducibility, particularly in patients with variable loading conditions. Recently, myocardial work (MW) parameters by PSL have been acknowledged as sensitive and comprehensive indicators for detecting subclinical LV myocardial dysfunction [6], assessing patient’s stratification, and estimating the prognosis in patients with different cardiovascular diseases. However, LV dysfunction in patients with OSAS has been seldom studied using MW parameters.

Therefore, the aims of the present study were 1) to assess LV performance using LV MW parameters by PSL technique in patients with OSAS having preserved LVEF and 2) to evaluate the relationship between MW parameters and OSAS severity.

## Methods

### Study population

In this study, we consecutively enrolled and recruited patients newly diagnosed with OSAS by full-night polysomnography (PSG) between February 2021 and December 2021 from the Respiratory Sleep Center of Beijing Chaoyang Hospital. All patients were divided into three groups according to the apnea–hypopnea index (AHI): mild group (5/h ≤ AHI < 15/h), moderate group (15/h ≤ AHI < 30/h), and severe group (AHI ≥ 30/h) [7]. All patients had a LVEF > 50% [8]. The exclusion criteria were as follows: central sleep apnea, prior treatment for OSAS, uncontrolled systemic hypertension, atrial fibrillation or any arrhythmia, coronary heart disease (identified by patient history, questionnaires, symptoms of angina or equivalent symptoms, electrocardiography, or echocardiography), history of congestive heart failure, moderate to severe valvular heart disease, pulmonary diseases, diabetes mellitus, chronic renal diseases (defined as the presence of abnormalities of kidney structure or function or an estimated glomerular filtration rate less than 60 ml/min per 1.73 square meters, persisting for more than 3 months [9]), history of malignancy, and dissatisfactory acoustic window. This study protocol was approved by the Ethics Committee of Chaoyang Hospital (No. 2021-K-592), and all participants provided written informed consent.

### Polysomnography

Epworth Sleepiness Scale score was used to access subjective daytime sleepiness and establish the probability of OSAS at first. All enrolled participants underwent overnight PSG using Embla N7000 (RemLogic Eastmed, Natus). Sleep study parameters were analyzed by experienced technicians according to the standard criteria. The following aspects were monitored and recorded: electroencephalogram (EEG), electrooculogram (EOG), chin electromyogram (EMG), oral and nasal airflow, thoracal and abdominal movement sensors, two leg movement sensors, body position detector, tracheal sound, electrocardiogram (ECG), and blood oxygen saturation. Apnea was defined as complete cessation of airflow lasting at least 10 s. Hypopnea was defined as > 30% reduction of respiratory airflow lasting at least 10 s accompanied by a decrease of 3% in oxygen saturation or EEG microarousal [10]. The apnea–hypopnea index (AHI) was defined as the number of apnea and/or hypopnea episodes per hour of sleep. OSAS was diagnosed if the AHI revealed ≥ 5 episodes per hour. The mean saturation of arterial oxygen (mean SaO_2_), minimum saturation of arterial oxygen (minimal SaO_2_), oxygen desaturation index, and percentage of sleep time with oxygen saturation below 90% (SP90) were also evaluated and recorded.

### Echocardiography

Two-dimensional transthoracic echocardiographic and Doppler studies were performed using a GE Vivid E95 ultrasound system (Vingmed Ultrasound, Horten, Norway) with M5S 3.5-MHz transducers. Patients were imaged at rest in supine and left lateral positions, and blood pressure was recorded simultaneously with the patient in the imaging position. Two-dimensional parameters, namely the LV internal dimension diastole (LVIDd), interventricular septal dimension (IVSd), and posterior wall dimension (PWTd), were measured in accordance with American Society of Echocardiography guideline (2015) [11]. Then, the LV mass (LVM) was calculated using the Devereux formula: LVM (g) = 0.8 × [1.04 (LVIDd + IVSd + PWTd)^3^-(LVIDd)^3^] + 0.6 and was normalized by body surface area (BSA). The LV end-diastolic volume (LVEDV) and LV end-systolic volume (LVESV) were measured using the modified Simpson’s rule from apical four- and two-chamber views, and the LVEF was calculated [9]. The LVEDV and the LVESV were divided by BSA to generate the LVEDV index and the LVESV index. Early peak transmitral flow velocity (E) and late peak atrial systolic velocity (A) were quantified using pulsed-wave Doppler echocardiography, and the E/A ratio was calculated and recorded. Lateral and septal early diastolic mitral annular velocities were obtained via tissue Doppler imaging from an apical four-chamber view, and their mean value (e’) and E/e’ ratio were subsequently calculated. Body mass index (BMI) and BSA was calculated as follows: BMI (kg/m^2^) = Weight (kg)/Height (m)^2^; BSA (m^2^) = ([Height (cm) × Weight (kg)]/3600)^1/2^. The systolic pulmonary arterial pressure was estimated based on the right atrial pressure taking into account the peak tricuspid regurgitation velocity using the simplified Bernoulli equation [12].

### 2D-STE and non-invasive PSL

**Fig. 1:**
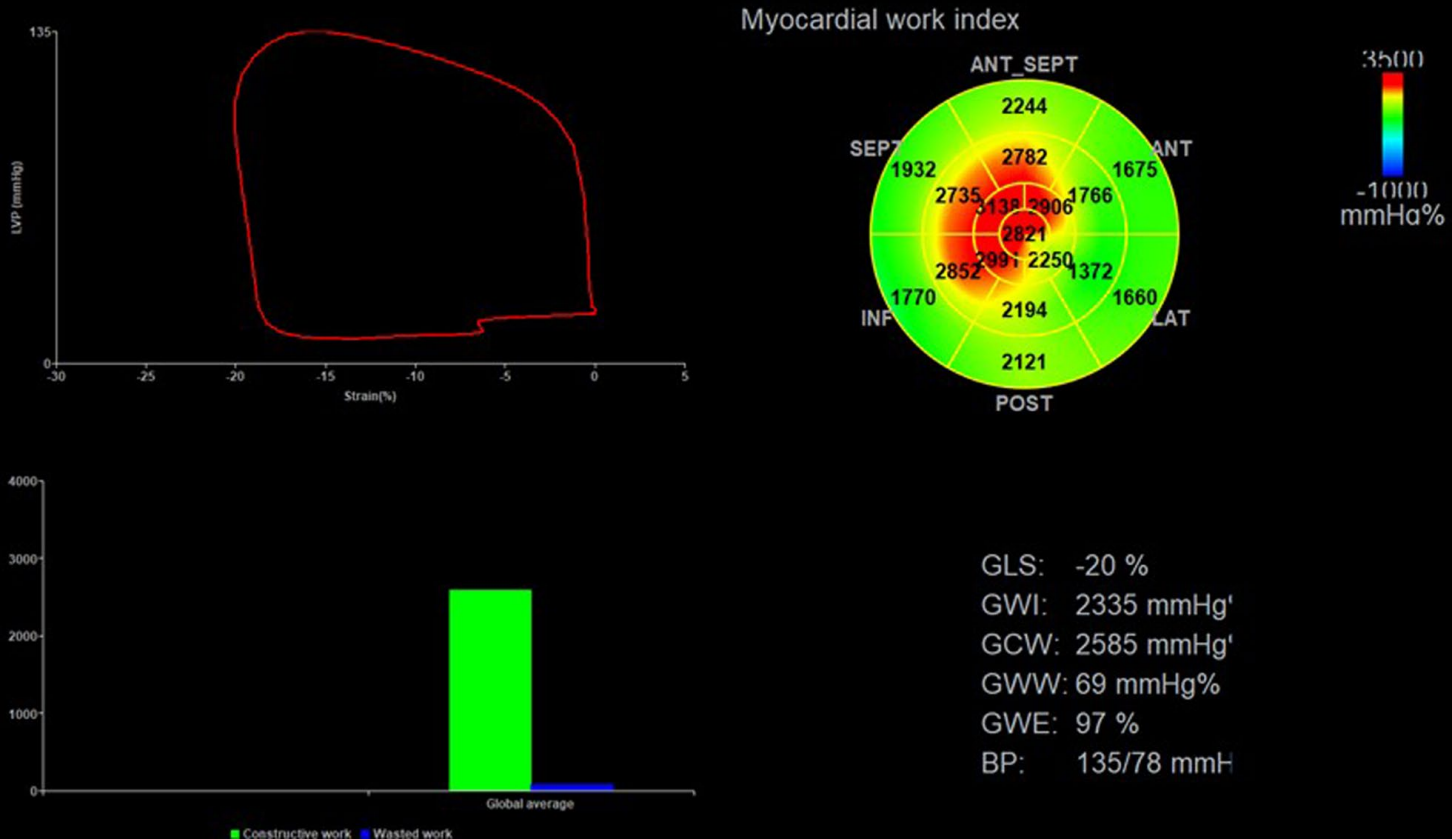
LV PSL was used to assess global and segmental GWI in patient with OSAS. PSL, pressure-strain loop; GLS, global longitudinal strain; GWI, global work index; GCW, global constructive work; GWW, global wasted work; GWE, global work efficiency, BP, blood pressure

All dynamic images were obtained over three consecutive cycles at a frame rate of ≥ 60/s and were stored in the EchoPAC software (Version 2.0.4, GE Vingmed Ultrasound, Norway) for offline analysis by three experienced doctors who were blinded to PSG findings. Images of apical four-, three-, and two-chamber views were stored. The LV global longitudinal strain was evaluated with automated function imaging on the three standard apical views. The endocardium was automatically tracked by the software. The width of the region of interest was adjusted if the LV wall thickness was not entirely included. The left ventricle was divided into 17 myocardial segments. GLS was calculated by averaging the peak longitudinal strain from the 17 segments and represented as an absolute value. MW was evaluated in the “Myocardial Work” mode with the same software (Fig. 1). Brachial cuff blood pressure, representing the pressure of the left ventricle, was entered into the software after GLS analysis [13].Subsequently, valvular event timing (including mitral valve closure, aortic valve opening, aortic valve closure, and mitral valve opening) were visually set by observing Doppler flow velocities across the mitral and aortic valves. A non-invasive PSL was constructed on the basis of blood pressure, valvular event times, and GLS. The global work index (GWI) corresponding to the area within the LV PSL was the total work performed by LV between mitral valve closure and mitral valve opening. Other parameters of MW were also included: Global constructive work (GCW) was defined as the positive work contributing to LV ejection and comprised the work done by the myocardium during LV shortening in systole and during elongation in isovolumic diastole. Global wasted work (GWW) was defined as the negative work against LV ejection and comprised the work done by the myocardium during LV elongation in systole and during shortening in isovolumic diastole. Global work efficiency (GWE) was calculated as constructive work divided by the sum of constructive and wasted work [14].

### Intra- and interobserver variability

Fifteen patients were randomly selected and measurements were repeated by the same operator at least a month later to assess the intraobserver variability. The interobserver variability was independently tested by a second experienced operator who was blinded to the results of the first operator.

### Statistical analysis

All data analyses were performed using SPSS version 25.0 (SPSS, Inc. Chicago, IL). *P*-values < 0.05 were considered statistically significant. All data were tested for normality and homogeneity of variance. Normally distributed continuous variables were expressed as the mean ± standard deviation, whereas non-normally distributed data were expressed as the median and interquartile range. Categorical variables were presented as frequencies and percentages. Between-group comparisons were performed using the one-way analysis of variance test with the Bonferroni post-hoc test for the normally distributed continuous data with equal variances. Tamhane's T2 was performed for comparison of normally distributed data with unequal variances. Kruskal–Wallis test was used for the comparison of non-normally distributed continuous data. The chi-square test was used for categorical variables. Pearson’s or Spearman’s correlation coefficients were used as appropriate for testing correlations of MW parameters with traditional echocardiographic measurements or PSG parameters. Only the parameters identified as having statistical significance (*P* < 0.05) on univariable linear regression were included in the multivariate linear regression model to identify independent predictors of each MW parameters in patients with OSAS. Intraclass correlations were studied to assess intra- and interobserver variability.

## Results

### General characteristics

Two hundred consecutive patients were finally included and were stratified by AHI. Based on the severity of OSAS, patients were divided into three groups: mild group (n = 50), moderate group (n = 55), and severe group (n = 95). General characteristics are shown in Table 1. Most patients were men (78.5%), and the overall mean age of patients was 42.0 ± 9.8 years. There was no significant difference in age, heart rate, or the use of ACEI/ARB, beta-blockers, and diuretics among the three groups (all, *P* > 0.05). However, sex, history of smoking, and history of hypertension significantly differed among groups (all, *P* < 0.05). BMI and BSA were significant different among groups and increased with the severity of the OSAS (*P* < 0.001). SBP was significantly higher in the severe group than in mild group (*P* < 0.001), and diastolic blood pressure was significantly lower in the severe group than in the mild group (*P* < 0.001) and the moderate group (*P* < 0.01).

**Table 1:** General characteristics of patients with OSAS

Variables	Total n = 200	Mild OSAS n = 50	Moderate OSAS n = 55	Severe OSAS n = 95	*P-*value
Male sex, n (%)	157 (78.5)	27 (54.0)	44 (80.0)^†^	86 (90.5)^†^	< 0.001
Age (years)	42.0 ± 9.8	41.5 ± 11.7	41.9 ± 10.6	42.2 ± 8.3	0.929
BMI (kg/m^2^)	26.3 ± 3.7	23.8 ± 3.3	25.9 ± 2.9^†^	27.8 ± 3.5^†^^,^^‡^	< 0.001
BSA (m^2^)	1.89 ± 0.21	1.74 ± 0.19	1.88 ± 0.19^†^	1.97 ± 0.19^†^^,‡^	< 0.001
Heart rate (BPM)	73.8 ± 12.2	71.4 ± 10.8	72.4 ± 10.5	75.8 ± 13.5	0.075
SBP (mmHg)	127.5 ± 14.1	121.1 ± 12.3	127.0 ± 12.5	131.2 ± 14.7^†^	< 0.001
DBP (mmHg)	78.6 ± 11.5	74.3 ± 8.6	76.7 ± 10.0	82.1 ± 12.3 ^†^^,‡^	< 0.001
Smoking, n (%)	78 (39.0)	7 (14.0)	21 (38.2) ^†^	50 (52.6) ^†^	< 0.001
Hypertension, n (%)	59 (29.5)	8 (16.0)	13 (22.4)	41 (43.2) ^†^^,‡^	< 0.001
Medications					
ACEI/ARB, n (%)	13 (6.5)	4 (8.0)	0 (0.0)	9 (9.5)	0.384
Beta-blockers, n (%)	9 (4.5)	1 (2.0)	2 (3.6)	6 (6.3)	0.460
CCB, n (%)	21 (10.5)	4 (8.0)	1 (1.8)	16 (16.8) ^†^	0.012
Diuretics, n (%)	4 (2.0)	1 (2.0)	0 (0.0)	3 (3.2)	0.412

*BMI* body mass index, *BSA* body surface area, *SBP* systolic blood pressure, *DBP* diastolic blood pressure, *ACEI/ARB* angiotensin-converting enzyme inhibitor/angiotensin receptor blockers, *CCB* calcium channel blockers

^†^*P* < 0.05, significantly different from Mild OSAS

^‡^*P* < 0.05, significantly different from Moderate OSAS

### Polysomnographic parameters

The polysomnographic variables are listed in Table 2. Compared with the mild group, moderate and severe groups showed significantly higher AHI, SP90, and Epworth Sleepiness Scale score and significantly lower mean saturation of arterial oxygen and minimum saturation of arterial oxygen (all, *P* < 0.001).

**Table 2:** Sleep study data of patients with OSAS

Variables	Total n = 200	Mild OSAS n = 50	Moderate OSAS n = 55	Severe OSAS n = 95	*P-*value
AHI (/h)	28.7 (14.9–52.0)	8.2 (6.2–12.1)	22.7 (19.0–26.1)^†^	52.4 (42.8–65.8)^†^^,‡^	< 0.001
Mean SaO_2_ (%)	93.4 ± 2.8	95.0 ± 1.2	94.2 ± 1.9^†^	92.0 ± 3.1^†^^,‡^	< 0.001
Minimal SaO_2_ (%)	79.4 ± 9.5	87.2 ± 3.4	82.7 ± 4.6^†^	73.1 ± 9.8^†^^,‡^	< 0.001
SP90 (%)	2.0 (0.1–7.3)	0.1 (0.0–0.4)	1.5 (0.1–4.6) ^†^	6.5 (1.8–23.4)^†^^,‡^	< 0.001
ESS score	8.5 ± 4.9	7.5 ± 4.6	9.1 ± 5.6	9.2 ± 4.8	0.091

*AHI* apnea–hypopnea index, *Mean SaO*_*2*_ mean saturation of arterial oxygen, *minimal SaO*_*2*_ minimum saturation of arterial oxygen, *SP90* percentage of sleep time with oxygen saturation below 90%, *ESS* Epworth Sleepiness Scale

^†^*P* < 0.05, significantly different from mild OSAS

^‡^*P* < 0.05, significantly different from moderate OSAS

### Standard echocardiographic parameters

The standard echocardiographic parameters for all groups are shown in Table 3. LVIDd was greater in moderate and severe groups than in the group (*P* < 0.001). IVSd and PWTd were higher in the severe group than in mild and moderate groups and were higher in the moderate group than in mild group. (*P* < 0.001). The LV mass index (LVMI) was significantly higher in moderate and severe groups than in the mild group (*P* < 0.001). The value of LVEDV index, LVESV index and LVEF did not significantly differ among groups (*P* > 0.05). However, the values of mitral E/e’ were significantly higher in the severe group than in mild group (*P* < 0.05). E/A was significantly lower in the severe group than in mild group (*P* < 0.05). The value of e’ was significantly lower in the severe group than in the mild group (*P* < 0.001) and the moderate group (*P* = 0.001) and was lower in the moderate group than in the mild group (*P* < 0.05). The value of systolic pulmonary arterial pressure did not significantly differ among groups (*P* > 0.05).

**Table 3:** Conventional echocardiographic parameters of patients with OSAS

Variables	Total n = 200		Mild OSAS n = 50	Moderate OSAS n = 55	Severe OSAS n = 95	*P-*value
LVIDd (mm)	46.8 ± 3.4		45.0 ± 3.3	47.1 ± 3.3 ^†^	47.5 ± 3.3 ^†^	< 0.001
IVSd (mm)	10.0 ± 1.1		9.2 ± 0.9	9.9 ± 1.1^†^	10.4 ± 1.0^†^^,‡^	< 0.001
PWTd (mm)	9.6 ± 1.1		8.7 ± 0.9	9.5 ± 1.1^†^	10.1 ± 1.1^†^^,‡^	< 0.001
LVEDV index (mL/m^2^)		61.8 ± 10.1	60.2 ± 8.6	61.9 ± 10.5	63.1 ± 10.5	0.332
LVESV index (mL/m^2^)		20.9 ± 5.6	19.3 ± 3.7	20.9 ± 6.8	21.5 ± 5.6	0.107
LVEF (%)	67.2 ± 3.9		68.1 ± 3.2	67.0 ± 4.2	66.9 ± 3.9	0.213
LVMI (g/m^2^)	84.7 ± 15.4		76.0 ± 12.1	85.0 ± 15.3^†^	89.3 ± 15.9^†^	< 0.001
Mitral E/e’	8.2 ± 1.8		7.6 ± 1.7	8.1 ± 1.7	8.5 ± 1.8^†^	0.022
E/A	1.2 (0.9–1.4)		1.2 (0.9–1.4)	1.3 (1.1–1.5)	1.2 (0.9–1.3)^†^	0.017
e’(cm/s)	10.3 ± 2.1		11.4 ± 2.3	10.7 ± 1.9^†^	9.5 ± 1.8^†^^,‡^	< 0.001
sPAP(mmHg)*	24.0 (21.0–28.7)		24.0 (20.0–30.0)	24.0 (20.8–28.5)	24.5 (22.0–28.0)	0.750

*LVIDd* left ventricular internal dimension diastole, *IVSd* interventricular septal dimension, *PWTd* posterior wall dimension, *LVEDV* left ventricular end-diastolic volume, *LVESV* left ventricular end-systolic volume, *LVEF* left ventricular ejection fraction, *LVMI* left ventricular mass index, *Mitral E/e’* the ratio of the early peak mitral flow velocity to e’, *E/A* the ratio of the early peak transmitral flow velocity to the late peak atrial systolic velocity, *e’* the average of lateral and septal early diastolic mitral annular velocity, *sPAP* systolic pulmonary arterial pressure

^†^*P* < 0.05, significantly different from mild OSAS

^‡^*P* < 0.05, significantly different from moderate OSAS

*Only 161 patients had tricuspid regurgitation velocity data and calculated sPAP. There were 43 patients, 46 patients and 72 patients in the mild group, moderate group and severe group, respectively

### MW parameters and GLS in patients with OSAS

**Fig. 2:**
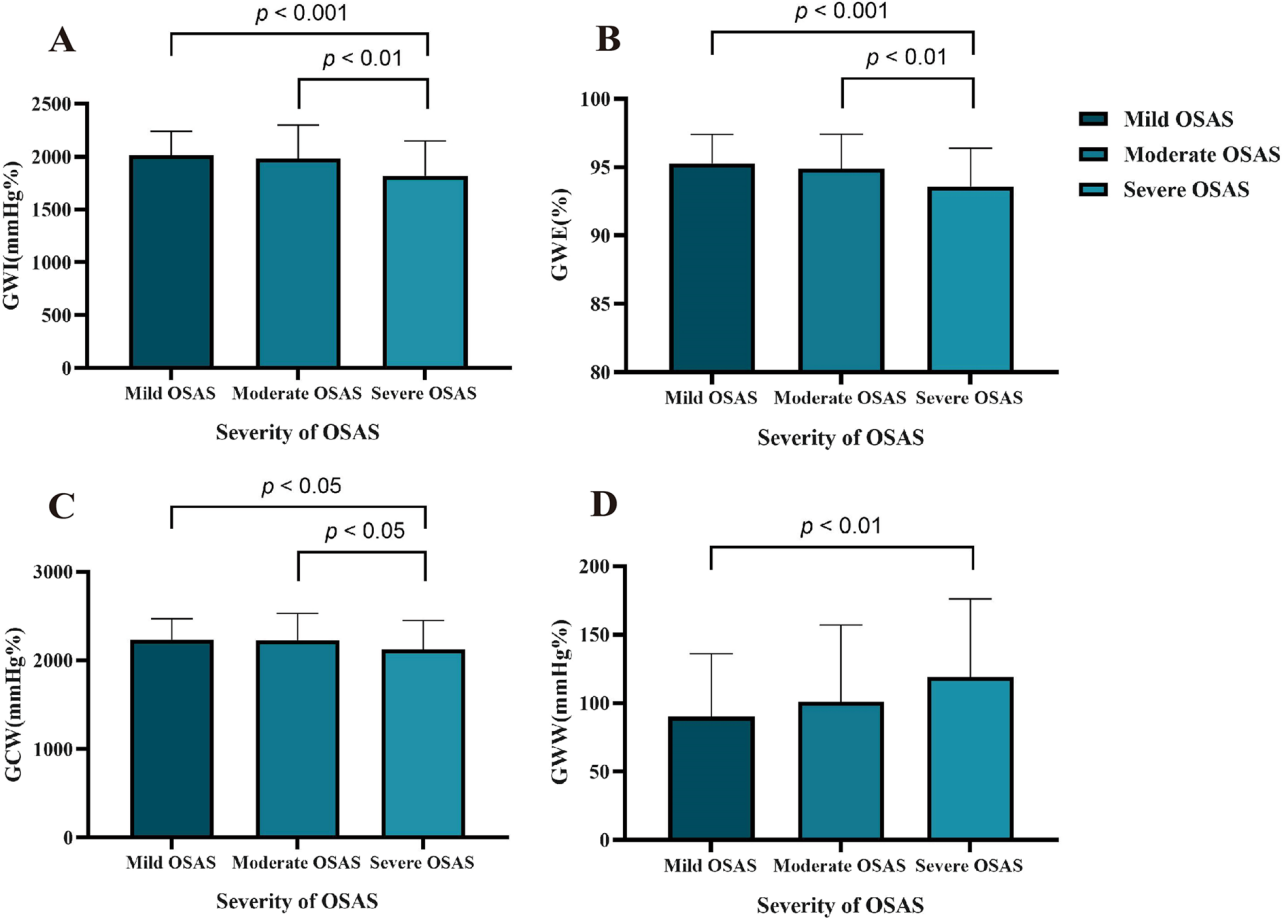
Comparison of myocardial work parameters among different groups in patients with OSAS. **A** GWI was significantly lower in the severe group than in the mild group and the moderate group. **B** GWE was significantly lower in the severe group than in the mild group and the moderate group. **C** GCW was significantly lower in the severe group than in the mild group and the moderate group. **D** GWW in the severe group was significantly higher than in the mild group

LV GLS and global MW in patients with OSAS are shown in Table 4. The value of LV GLS in patients with OSAS was significantly lower in the severe group than in mild and moderate groups (all, *P* < 0.001). GWI was significantly lower in the severe group (1820.2 ± 328.2 mmHg%) than in the mild group (2013.1 ± 225.3 mmHg%, *P* < 0.001) and the moderate group (1982.3 ± 316.4 mmHg%, *P* < 0.01, Fig. 2A). GWE was significantly lower in the severe group than in the mild group [96.0% (94.0%–97.0%) *vs*. 94.0% (92.0%–95.0%), *P* < 0.001] and the moderate group [96.0% (94.0%–97.0%) *vs*. 94.0% (92.0%–95.0%), *P* < 0.01; Fig. 2B]. GCW was significantly lower in the severe group than in the mild group (2120.0 ± 330.5 mmHg% *vs*. 2233.2 ± 235.7 mmHg%, *P* < 0.05) and the moderate group (2120.0 ± 330.5 mmHg% *vs*. 2227.6 ± 301.4 mmHg%, *P* < 0.05, Fig. 2C). GWW in the severe group [115.0 (IQR: 81.0–156.0) mmHg%] was significantly higher than in the mild group [77.5 (IQR: 57.8–108), *P* < 0.01] but insignificantly lower than in the moderate group [87.0 (IQR: 63.0–133.0),* P* > 0.05; Fig. 2D].

**Table 4:** Myocardial work parameters of patients with OSAS

Variables	Total n = 200	Mild OSAS n = 50	Moderate OSAS n = 55	Severe OSAS n = 95	*P-*value
GLS (%)	19.2 ± 2.4	20.6 ± 2.2	19.7 ± 2.2	18.2 ± 2.2^†^^,‡^	< 0.001
GWI (mmHg%)	1913.0 ± 314.0	2013.1 ± 225.3	1982.3 ± 316.4	1820.2 ± 328.2^†^^,‡^	< 0.001
GWE (mmHg%)	95.0 (93.0–96.0)	96.0 (94.0–97.0)	96.0 (94.0–97.0)	94.0 (92.0–95.0)^†^^,‡^	< 0.001
GCW (mmHg%)	2177.8 ± 304.9	2233.2 ± 235.7	2227.6 ± 301.4	2120.0 ± 330.5^†^^,‡^	0.040
GWW (mmHg%)	92.5 (69.0–134.5)	77.5 (57.8–108)	87.0 (63.0–133.0)	115.0 (81.0–156.0) ^†^	0.002

*GLS* global longitudinal strain, *GWI* global work index, *GWE* global work efficiency, *GCW* global constructive work, *GWW* global wasted work

^†^*P* < 0.05, significantly different from mild OSAS

^‡^*P* < 0.05, significantly different from moderate OSAS

### MW parameters and the severity of OSAS

Table 5 shows the results of correlation analysis between systolic function parameters and OSAS severity. GLS, GWI, and GWE showed a moderate correlation with AHI, with Spearman’s ρ of −0.468, −0.321, and −0.319, respectively (*P* < 0.001). GCW and GWW were weakly correlated with AHI (Spearman’s ρ = −0.226 and 0.255, *P* < 0.001, respectively). However, LVEF was not associated with AHI (*P* = 0.089). Multiple regression analyses revealed that SBP (β = 0.733, *P* < 0.001), GLS (β = 0.717, *P* < 0.001), e’ (β = 0.103, *P* = 0.016), and AHI (β = −0.155 *P* < 0.001; Table 6) were independently correlated with GWI. SBP (β = 0.802, *P* < 0.001), GLS (β = 0.769, *P* = 0.013), and AHI (β = −0.082, *P* = 0.037; Table 6) were independently associated with GCW. However, only GLS (β = 0.421, *P* < 0.001) and BMI (β = −0.236, *P* = 0.002; Additional file 1: Table S1) were independently associated with GWE. Similarly, only GLS (β = −0.294, *P* < 0.001) and BMI (β = 0.241, *P* = 0.002; Additional file 2: Table S2) were independently associated with GWW.

**Table 5:** Correlation between AHI and systolic parameters

Variables	AHI	
	Spearman’s ρ	*P-*value
GLS	−0.468	< 0.001
GWI	−0.321	< 0.001
GWE	−0.319	< 0.001
GCW	−0.226	< 0.001
GWW	0.255	< 0.001
LVEF	−0.121	0.089

*GLS* global longitudinal strain, *GWI* global work index, *GWE* global work efficiency, *GCW* global constructive work, *GWW* global wasted work, *LVEF* left ventricular ejection fraction

**Table 6:** Univariable and multivariable linear regression analysis of GWI and GCW

Variables	GWI				GCW			
	Univariable analysis		Multivariable analysis		Univariable analysis		Multivariable analysis	
	*β*-coefficient	*P-*value	*β*-coefficient	*P-*value	*β*-coefficient	*P-*value	*β*-coefficient	*P-*value
SBP	0.370	< 0.001	0.733	< 0.001	0.463	< 0.001	0.802	< 0.001
BMI	−0.193	0.006	0.055	0.263	−0.153	0.030	0.022	0.610
Age	0.077	0.276			0.124	0.080		
Male gender	0.187	0.008	0.029	0.469	−0.154	0.030	0.018	0.637
Heart rate	−0.198	0.005	−0.019	0.629	−0.181	0.010	−0.030	0.412
Smoking	−0.143	0.043	−0.012	0.773	−0.121	0.087		
ACEI/ARB	−0.040	0.574			0.034	0.634		
Beta-blockers	0.105	0.140			0.138	0.051		
CCB	−0.024	0.737			0.044	0.536		
Diuretics	−0.156	0.028	−0.010	0.794	−0.134	0.058		
GLS	0.546	< 0.001	0.717	< 0.001	0.472	< 0.001	0.769	< 0.001
Mitral E/e’	0.133	0.061			0.164	0.061		
e’	0.251	< 0.001	0.103	0.016	0.153	0.031	0.051	0.191
E/A	0.147	0.038	0.031	0.483	0.059	0.406		
LVMI	0.106	0.134			0.182	0.010	0.046	0.226
AHI	−0.284	< 0.001	−0.155	< 0.001	−0.190	0.007	−0.082	0.037
ESS score	−0.174	0.013	−0.002	0.957	−0.179	0.011	−0.003	0.965

*GWI* global work index, *GCW* global constructive work, *β* standardized regression coefficients, *SBP* systolic blood pressure, *BMI* body mass index, *ACEI/ARB* angiotensin-converting enzyme inhibitor/angiotensin receptor blockers, *CCB* calcium channel blockers, *GLS* global longitudinal strain, *Mitral E/e’* the ratio of the early peak mitral flow velocity to e’, *e’* the average of lateral and septal early diastolic mitral annular velocity, *E/A* the ratio of the early peak transmitral flow velocity to the late peak atrial systolic velocity, *LVMI* left ventricular mass index, *AHI* apnea–hypopnea index, *ESS* Epworth Sleepiness Scale

### Intra- and interobserver variability for MW indices

The results of the intra- and interobserver variability for MW parameters are shown in Table 7. All MW parameters exhibited good intra- and interobserver correlation, with intraclass correlation coefficient values > 0.80.

**Table 7:** Intra- and interobserver variability for myocardial work parameters

Variables	Intra-observer		Inter-observer	
	ICC	95% CI	ICC	95%CI
GWE	0.80	0.50–0.93	0.81	0.53–0.93
GWI	0.97	0.89–0.99	0.97	0.92–0.99
GCW	0.98	0.95–0.99	0.98	0.92–0.99
GWW	0.90	0.75–0.97	0.86	0.65–0.95

*ICC* intraclass correlation coefficient, *CI* confidence interval, *GWE* global work efficiency, *GWI* global work index, *GCW* global constructive work, *GWW* global wasted work

## Discussion

OSAS is independently associated with cardiovascular diseases, such as arrythmias and heart failure. Myocardial damage at early stage is less severe and thus reversible [15]. Therefore, early identification of myocardial injury and timely cardioprotective intervention are of great significance. In the present study, we assessed subclinical LV myocardial dysfunction in patients with OSAS having preserved LVEF using MW parameters. We found that GWI, GCW, and GWE were decreased while GWW was increased in the severe group compared to the mild and moderate groups in OSAS patients with preserved LVEF. In addition, MW indices were correlated with the severity of OSAS. Finally, increased AHI was independent associated with both reduced GWI and GCW.

### Mechanism for myocardial injury related to OSAS

Mechanics underlying systolic dysfunction in OSAS are complicated and not elucidated entirely thus far. Intermittent hypoxia is well known to have a role in cardiac dysfunction, and it can cause LV remodeling and dysfunction [16]. Prolonged overactivation of the sympathetic nervous system raises the oxygen demand and leads to interstitial fibrosis by elevating the systemic blood pressure, increasing the heart rate, and generating negative intrathoracic pressure [17–19]. Systemic inflammation [20], oxidative stress [21], activation of renin–angiotensin–aldosterone system [22, 23] are other deleterious effects that result in dysfunction of the vascular endothelium, interstitial fibrosis, and progressive cardiac remodeling [24, 25].

### Alteration in conventional 2D-STE parameters

GLS, a widely used measure and a sensitive parameter of myocardial dysfunction with established prognostic value, can help identify subtle changes in early systolic dysfunction when LVEF is preserved. A number of echocardiographic studies have been done to detect the early change of myocardial function in patients with OSAS using 2D-STE [26]. Zhou et al. showed that the longitudinal strain was significant lower in the severe group when compared with controls, mild group and moderate group, and the strain was significantly correlated with the degree of OSAS [3]. Likewise, Altekin et al. found the longitudinal strain decreased as the severity of disease increased from moderate towards severe OSAS [27]. In contrast to previous research, the GLS in our study was not different between mild and moderate groups. This may because we did not have healthy people as the control group, so the mild group was the reference group. This may also attribute to the excessively skewed distribution of AHI and the inclusion of hypertensive patients, resulting in the significant lower GLS in the severe group. Nevertheless, we found that the severe group was associated with a lower absolute GLS value than the mild or moderate group and the absolute GLS value was negatively correlated with AHI, which corroborates previous research.

Load is an important factor that impacts parameters quantifying LV systolic function. However, GLS only reflects the relative length change and does not account for afterload [11]. This is a limitation encountered while investigating patients with OSAS because their hemodynamic conditions change easily [22]. Prior studies have shown that OSAS is highly prevalent in hypertensive patients, among whom 30%–50% have comorbid OSAS [28]and the risk of essential hypertension increases with increasing severity of OSAS [29, 30]. Some studies proved that blood pressure decreased in normotensive individuals even after continuous positive airway pressure (CPAP) [31].

### Relationship between myocardial work and severity of OSAS

Unlike GLS, which only reflects peak systolic strain and can be influenced by LV loading conditions, PSL calculates the work of myocardium incorporating stress load and myocardial deformation during the cardiac cycle [32]. Non-invasive PSL area has been validated against invasively measured pressure–volume area in an experimental study [14]. Moreover, a study evaluating myocardial function from perspective of cardiac metabolic demand reported that GWI has a good correlation with myocardial glucose metabolism as measured by positron emission tomography (PET) [32]. MW indices have proven to be more sensitive when compared with traditional methods and have diagnostic value in a range of diseases [33–35]. As shown in our data, MW parameters detected changes in subclinical LV systolic function in our study. Compared with the mild OSAS group, GWI, GCW and GWE were decreased and GWW was increased in the severe group.

Severity of OSAS showed an independent association with both GWI and GCW. Repetitive hypoxia disturbs the delicate balance of myocardial oxygen supply and demand [26], thus resulting in the interstitial fibrosis and microvascular endothelium injury [24, 25]. These changes consequently lead to the deterioration of myocardial energy exploitation and metabolic activity, which may explain the reduction of GWI and GCW. Huang et al. found that GWI and GCW were lower in patients with diabetes mellitus after adjusting for blood pressure and clinical parameters [36]. Moreover, GWI and GCW were reportedly higher in patients with Turner syndrome than in healthy individuals even though blood pressure and GLS were not significantly different [37]. Interestingly, AHI was independent associated with both GWI and GCW after adjusting for GLS in our study. Taken together, PSL technology can be considered a more sensitive and comprehensive parameter in evaluating early impairment of ventricular function.

In addition, systolic and diastolic functions are closely related [38]. As a result of inadequate compression and twisting of extracellular matrix during the systole, the loss of LV suction can induce abnormal relaxation and impaired diastolic function [39]. The value of e’ was independently associated with GWI in our study, which corroborates previous research [40]. The significant contribution of deformation in the calculation of MW may explain the independent associations between GLS and MW indices in our study [41]. Furthermore, SBP was not surprisingly proved to be an independent predictor of both GWI and GCW. Level of work was significantly increased in conditions of high blood pressure as a compensatory mechanism to preserve LV contractility against increased afterload [42].

### Limitations

This study has several limitations. First, the sample size in this study is relatively small, and the patients were enrolled from a single center. Only subjects with sleep disordered breathing symptoms underwent overnight PSG due to the facility shortage during our study period, resulting in limited data in the control group. We need to enroll normal subjects in the future. A further large-scale study is warranted to verify our results. Second, the generalizability of our findings remains to be determined. Patients with a cardiac, pulmonary, or renal disease were excluded from this study, and thus, our results cannot be extrapolated to these individuals. Third, we could not obtain details on some baseline characteristics, such as duration of OSA and duration of hypertension and hyperlipidemia. Last but not the least, there was a significant difference in the number of patients in each group, and the severe group is the largest. This may explain the insignificant differences in GLS and MW parameters between the mild and moderate groups, which is different from previous study including similar number of patients in each group.

## Conclusion

This study demonstrated that the LV PSL technique could be used to assess myocardial function in patients with OSAS in the early stage. Increased severity of OSAS was demonstrated to have an independent association with both decreased GWI and GCW. Future studies aimed at evaluating the prognostic impact of MW on cardiovascular outcomes in patients with OSAS are needed.

## Supplementary Information

**Additional file 1**.**Supplemental Table 1.** Univariable and multivariable linear regression analysis of GWE.

**Additional file 2**.**Supplemental Table 2.** Univariable and multivariable linear regression analysis of GWW.

## Data Availability

The datasets of this study are not publicly available because of the restrictions by the Beijing Chaoyang Hospital. The authors used this dataset under an agreement with the Beijing Chaoyang Hospital for the study. The data are available from the corresponding author on reasonable request.

## Abbreviations

PSL: Pressure–strain loop
OSAS: Obstructive sleep apnea syndrome
LV: Left ventricular
2D-STE: Two-dimensional speckle tracking echocardiography
LVEF: Left ventricular ejection fraction
PSG: Polysomnography
MW: Myocardial work
GLS: Global longitudinal strain
GWI: Global work index
GWE: Global work efficiency
GCW: Global constructive work
GWW: Global wasted work
BMI: Body mass index
BSA: Body surface area
SBP: Systolic blood pressure
ACEI/ARB: Angiotensin-converting enzyme inhibitor/angiotensin receptor blockers
CCB: Calcium channel blockers
AHI: Apnea–hypopnea index
Mean SaO_2_: Mean saturation of arterial oxygen, minimal SaO_2_: minimum saturation of arterial oxygen
SP90: Percentage of sleep time with oxygen saturation below 90%
ESS: Epworth Sleepiness Scale
LVIDd: Left ventricular internal dimension diastole
IVSd: Interventricular septal dimension
PWTd: Posterior wall dimension
LVEF: Left ventricular ejection fraction
LVMI: Left ventricular mass index
LVEDV: Left ventricular end-diastolic volume
LVESV: Left ventricular end-systolic volume
Mitral E/e’: The ratio of the early peak mitral flow velocity to e′
E/A: The ratio of the early peak transmitral flow velocity to the late peak atrial systolic velocity
e’: The average of lateral and septal early diastolic mitral annular velocity
