# Supplementary material for: Subclinical left ventricular myocardial dysfunction in patients with obstructive sleep apnea syndrome: insights from noninvasive left ventricular myocardial work analysis
Source: BMC Cardiovasc Disord. 2022 Dec 19;22:552. doi: 10.1186/s12872-022-03006-9 (PMC9761973; doi:10.1186/s12872-022-03006-9)
Supplement: Supplementary file 1 — Additional file 1.Supplemental Table 1. Univariable and multivariable linear regression analysis of GWE. [file 12872_2022_3006_MOESM1_ESM.docx]

**Supplementary material**

**Supplemental Table 1.** Univariable and multivariable linear regression analysis of GWE

| Variables | Univariable analysis | | | Multivariable analysis | |
| --- | --- | --- | --- | --- | --- |
|  | *β*-coefficient | | *P-*value | *β*-coefficient | *P-*value |
| SBP | -0.213 | | 0.002 | 0.082 | 0.231 |
| BMI | -0.469 | | <0.001 | -0.236 | **0.002** |
| Age | | -0.112 | 0.115 |  |  |
| Male gender | 0.116 | | 0.103 |  |  |
| Heart rate | -0.074 | | 0.295 |  |  |
| Smoking | -0.071 | | 0.317 |  |  |
| ACEI/ARB | -0.249 | | <0.001 | -0.051 | 0.478 |
| Beta-blockers | -0.220 | | 0.068 |  |  |
| CCB | -0.286 | | <0.001 | -0.116 | 0.076 |
| Diuretics | -0.274 | | <0.001 | -0.085 | 0.243 |
| GLS | 0.549 | | <0.001 | 0.421 | **<0.001** |
| Mitral E/e’ | -0.091 | | 0.201 |  |  |
| e' | 0.325 | | <0.001 | 0.009 | 0.907 |
| E/A | 0.208 | | 0.003 | 0.005 | 0.941 |
| LVMI | -0.197 | | 0.005 | -0.085 | 0.213 |
| AHI | -0.286 | | <0.001 | 0.059 | 0.413 |
| ESS score | -0.082 | | 0.247 |  |  |

Abbreviations: GWE, global work efficiency; β, standardized regression coefficients; SBP, systolic blood pressure; BMI, body mass index; ACEI/ARB: angiotensin-converting enzyme inhibitor/angiotensin receptor blockers; CCB, calcium channel blockers; GLS, global longitudinal strain; Mitral E/e’, the ratio of the early peak mitral flow velocity to e’; e’, the average of lateral and septal early diastolic mitral annular velocity; E/A, the ratio of the early peak transmitral flow velocity to the late peak atrial systolic velocity; LVMI, left ventricular mass index; AHI, apnea–hypopnea index; ESS, Epworth Sleepiness Scale
