# Supplementary material for: Subclinical left ventricular myocardial dysfunction in patients with obstructive sleep apnea syndrome: insights from noninvasive left ventricular myocardial work analysis
Source: BMC Cardiovasc Disord. 2022 Dec 19;22:552. doi: 10.1186/s12872-022-03006-9 (PMC9761973; doi:10.1186/s12872-022-03006-9)
Supplement: Supplementary file 2 — Additional file 2.Supplemental Table 2. Univariable and multivariable linear regression analysis of GWW. [file 12872_2022_3006_MOESM2_ESM.docx]

**Supplementary material**

**Supplemental Table 2.** Univariable and multivariable linear regression analysis of GWW

| Variables | Univariable analysis | | Multivariable analysis | |
| --- | --- | --- | --- | --- |
|  | *β*-coefficient | *P-*value | *β*-coefficient | *P-*value |
| SBP | 0.265 | <0.001 | 0.048 | 0.509 |
| BMI | 0.423 | <0.001 | 0.241 | **0.002** |
| Age | 0.137 | 0.054 |  |  |
| Male gender | 0.065 | 0.359 |  |  |
| Heart rate | 0.003 | 0.970 |  |  |
| Smoking | 0.065 | 0.359 |  |  |
| ACEI/ARB | 0.303 | <0.001 | 0.126 | 0.108 |
| Beta-blockers | 0.247 | <0.001 | 0.126 | 0.067 |
| CCB | 0.277 | <0.001 | 0.022 | 0.774 |
| Diuretics | 0.296 | <0.001 | 0.107 | 0.163 |
| GLS | -0.427 | <0.001 | -0.294 | **<0.001** |
| Mitral E/e’ | 0.093 | 0.192 |  |  |
| e’ | -0.250 | <0.001 | 0.066 | 0.417 |
| E/A | -0.173 | 0.014 | -0.012 | 0.867 |
| LVMI | 0.245 | <0.001 | 0.124 | 0.092 |
| AHI | 0.214 | 0.002 | -0.091 | 0.248 |
| ESS score | 0.020 | 0.781 |  |  |

Abbreviations: GWW, global wasted work; β, standardized regression coefficients; SBP, systolic blood pressure; BMI, body mass index; ACEI/ARB: angiotensin-converting enzyme inhibitor/angiotensin receptor blockers; CCB, calcium channel blockers; GLS, global longitudinal strain; Mitral E/e’, the ratio of the early peak mitral flow velocity to e’; e’, the average of lateral and septal early diastolic mitral annular velocity; E/A, the ratio of the early peak transmitral flow velocity to the late peak atrial systolic velocity; LVMI, left ventricular mass index; AHI, apnea–hypopnea index; ESS, Epworth Sleepiness Scale
